# Protected areas provide incomplete coverage for wintering Red Kites (*Milvus milvus*) in European agricultural landscapes

**DOI:** 10.1371/journal.pone.0355680

**Published:** 2026-08-26

**Authors:** Connor T. Panter, Pratik R. Gupte, Ana Bermejo-Bermejo, Javier de la Puente, Jorge García-Macía, Ivan Literák, Rainer Raab, Jan Škrábal, Arturo Rodríguez, Vicente Urios, Rachel L. White, Bryony A. Tolhurst

**Affiliations:** 1 School of Geography, University of Nottingham, University Park, Nottingham, United Kingdom; 2 School of Applied Sciences, University of Brighton, Brighton, United Kingdom; 3 Groningen Institute for Evolutionary Life Sciences, University of Groningen, Groningen, The Netherlands; 4 BirdWings Science and Conservation Consulting SL, El Escorial, Madrid, Spain; 5 Vertebrates Zoology Research Group, University of Alicante, Alicante, Spain; 6 Department of Biology and Wildlife Diseases, Faculty of Veterinary Hygiene and Ecology, University of Veterinary and Pharmaceutical Sciences Brno, Brno, Czech Republic; 7 Technisches Büro für Biologie Mag. Dr.Rainer Raab, Deutsch‐Wagram, Austria; 8 Asociacion Hontza, Zadorra Kale, Araba, Spain; University of Bucharest, ROMANIA

## Abstract

Designation and management of protected areas (PAs) can mitigate detrimental anthropogenic impacts on biodiversity. Across Europe, an extensive network of PAs exist to protect wildlife and habitats. These sites aim to provide important refugia for species of conservation interest, which include the Red Kite (*Milvus milvus*; hereafter “kites”). Currently, kites are classified as globally Least Concern but in danger of extinction in parts of their range such as Spain. However, it remains unclear to what extent kite ranges overlap with PAs with fundamental knowledge gaps persisting for the species during winter. Using data for 103 kites tagged with GPS-GSM telemetry loggers over 180 winters between 2013–2022, we modelled proportional overlap between kite winter ranges and both European Natura 2000 sites and Important Bird and Biodiversity Areas (IBAs). We also explored the effects of various demographic, geographic and environmental predictors of PA overlap in tagged kites. Overall, 24.5 ± 28.1% (± SD) of kite winter core areas and 26.6 ± 20.5% of winter home ranges overlapped with European PAs. Kites that wintered in areas with greater agricultural land cover had lower overlap with PAs. Kites that wintered in Iberia (Spain and Portugal) and the Pyrenees (France and into northern Spain) had lower overlap with PAs than conspecifics wintering in Central Europe. Wildlife that rely on traditional agricultural landscapes during winter may thus remain unprotected by existing European PAs. Policymakers should consider the conservation needs of such species when designating new PAs, especially as Spain holds the largest global wintering kite population, and the species is categorised as “In danger of extinction” in the Spanish National Catalogue of Threatened Species. Improving protection of wintering kites in Iberia remains an urgent conservation priority.

## Introduction

Biodiversity is declining rapidly due to human impacts including land use change, pollution, and climate change [1,2]. One key strategy to address these issues is the establishment and management of protected areas (hereafter “PAs”) to conserve biodiversity [3]. The creation of PAs, such as nature reserves and national parks, increased significantly in the latter half of the 20th century in response to habitat and species declines [4]. PAs remain a crucial tool for conserving land and protecting biodiversity from environmental change [5–7].

In response to the adoption of the Rio de Janeiro Convention on Biological Diversity (CBD) in 1992 [8], the European Union (EU) established a network of PA sites termed “Natura 2000” [9]. The network includes two major site categories: Special Protection Areas (SPAs) and Special Areas for Conservation (SACs) [10]. SPAs comprise sites of conservation value for rare and threatened European birds designated under the Birds Directive (Directive 2009/147/EC) [11]. Whereas SACs protect plants, animals (except birds) and wildlife habitats of EU importance designated under the “Habitats Directive” (92/43/CEE) [12]. Latest estimates suggest that the Natura 2000 network covers approximately 18.6% of EU member states and all biogeographic regions of Europe [13]. In non-EU countries such as Switzerland, similar conservation objectives are addressed through frameworks such as the Emerald Network, although these are not directly equivalent to Natura 2000 in legal structure or implementation. Simultaneously, a network of PAs exists to protect habitats essential for particular taxonomic groups [14], including Birdlife International’s Important Bird Areas programme [15]. In 2014, the terminology changed to “Important Bird and Biodiversity Areas” (IBAs) encapsulating the importance of these sites to wider biodiversity [16]. More than 13,000 IBA sites have been designated, comprising the most extensive network of PAs globally [15]. Although these networks do not encompass all PAs across Europe, including those designated under IUCN categories Ia–VI, Natura 2000 and IBAs represent the most extensive and standardised pan-European frameworks relevant to bird conservation. Their broad spatial coverage and consistent criteria make them particularly suitable for large-scale comparative analyses of species distributions and PA overlap.

However, in contrast to Natura 2000 sites, IBAs do not inherently confer legal protection. Rather, they are identified using standardised, science-based criteria to highlight sites of high importance for bird conservation and are intended to inform national and international conservation planning but are not automatically incorporated into national legislation [15]. While many IBAs overlap partially or entirely with Natura 2000 sites or other nationally designated PAs, others remain without statutory protection. Consequently, IBAs represent a heterogeneous network in terms of legal status, ranging from fully protected sites to areas recognised solely for their conservation value without formal legislative backing. This distinction is important when evaluating PA effectiveness, as ecological use of IBAs by species may not necessarily reflect the benefits of formal protection but instead represent underlying habitat suitability or landscape context.

Acting as fundamental refugia for many wildlife species [17], PAs are important for migratory species and those that occupy higher trophic levels such as raptors [18,19]. As apex predators, many raptors, *i.e.,* species within the orders Accipitriformes, Cathartiformes, Falconiformes, Strigiformes, and Cariamiformes [20], are considered sentinels of wider ecosystem health and are often regarded as key ecological indicator species [21]. However, of the approximately 557 described raptor species globally [22], many are under pressure from habitat loss and direct/indirect persecution by humans, and therefore experience population declines across parts of their range [23]. The Red Kite (*Milvus milvus*) is one example.

The Red Kite (hereafter referred to as “kites”) is a European endemic breeding raptor [24,25], which is legally protected throughout its range under Annex I of the EU Birds Directive (Directive 2009/147/EC). Central and northern European kites migrate to southern France, the Iberian Peninsula [26], Italy [27], and eastern Greece [28,29], while southern European populations, including those in Iberia, are largely resident year-round. Some Central European kites are also becoming sedentary [30,31]. Kites experience persecution through poisoning due to their facultative scavenging habits [32–35]. They prefer human-modified landscapes such as managed forests and croplands, which are especially beneficial in winter [26,36–38]. However, agricultural landscapes are often underrepresented within PA networks, potentially creating a mismatch between PA coverage and the habitats most frequently used by kites. Kites hunt small mammals and search for carrion in open habitats, often roosting in woodland fragments near farmland [27,39,40]. Agricultural intensification and rodenticide use pose significant threats through secondary poisoning [41–43], and their size increases the risk of collisions with energy infrastructure [44–46]. Although the species' global conservation status improved from ‘Near Threatened’ (2018) to ‘Least Concern’ (2020) on the IUCN Red List [23], declines persist in some areas of its range, notably in Spain where the resident population is considered “In danger of extinction” by the Spanish National Catalogue of Threatened Species [25]. Importantly, the Spanish population increasingly relies on PAs [47], yet threats on the Iberian Peninsula affect both local and migratory kites.

Understanding whether kite ranges occur within PAs during winter is crucial for their conservation, especially since many kites spend up to eight months away from their breeding grounds. While some research exists on raptor species in eastern and southern Spain, such as Bonelli’s Eagle [14] and other raptors [18], studies on kites are limited. General studies indicate that younger kites use larger ranges and travel farther than adults during winter [26], possibly due to inexperience [48]. Sex differences in range size and movement patterns have also been noted, with males traveling farther than females [38]. According to ecological theories on space use, organisms typically occupy the smallest area containing necessary resources [49,50]. Thus, larger winter ranges may reflect lower resource availability or quality, whereas smaller ranges may indicate more concentrated or higher-quality resources. Under this assumption, effective PAs would be expected to encompass key resources, resulting in smaller individual ranges and greater proportional PA overlap.

For some other raptor species studied in Europe and elsewhere (*e.g.,* ten species in Spain) [18], there is evidence that PAs are effective, and thus habitat quality is indeed high. However, for others, such as Bonelli’s Eagle and Lesser Kestrel in Spain, and Montagu’s Harriers (*Circus pygargus*) in the Sahel, inadequate coverage of suitable habitat suggests that PA designations are insufficient for safeguarding regional populations [14,18,51]. Further, analyses of avian electrocutions surrounding SPAs in Spain found inadequacies in the ability to sufficiently protect birds from anthropogenic infrastructure within them, contributing to deleterious edge-effects [52]. Such evidence from the Iberian Peninsula suggests that PAs in this region are potentially less effective than those in Northern or Central Europe. However, this pattern may simply reflect a bias in recording towards Spanish raptor populations.

The use of PAs by wintering kites and other raptors is clearly impacted by extrinsic factors such as land use within and surrounding PAs. Wintering kite selection of agricultural areas [36,37] indicates that PAs covering substantial proportions of agricultural habitats would therefore support healthy kite populations, however, human-modified habitats tend to be overlooked during the designation of new PAs [53]. In addition, fertile soils and agricultural habitats tend to be distributed throughout areas of low elevation [54] and there is ample evidence that winter kite distributions are restricted between 400–650 metres above sea level (m ASL) [27,37,47,55]. Thus, they are expected to strongly overlap.

In this study, we quantified the use of PAs, specifically Natura 2000 sites and IBAs, by wintering kites throughout the ranges of tagged individuals. We focused on these PA types as they provide extensive and widely applied coverage across Europe [13], allowing consistent comparisons across regions. While we acknowledge that other nationally designated or IUCN-classified protected areas are not included, Natura 2000 and IBAs offer a harmonised framework suitable for large-scale analyses of species–PA relationships. Using telemetry data, we explored the effects of intrinsic (sex, age, and range size) and extrinsic (land use, elevational, and regional) factors predicting proportional PA overlap with kite winter ranges. We expected to find: 1) a negative correlation between absolute kite winter range size and proportional overlap with PAs, *i.e.,* larger ranges contain less overlap; 2) a positive effect of kite age on PA overlap with winter ranges, *i.e.,* adults overlap PAs more than younger birds; 3) a sex-bias in PA winter kite range overlap, where males overlap less than females; 4) a lower PA/winter range overlap by Iberian kites compared to other regions; and 5) a negative effect of lowland agricultural land cover on PA/range overlap.

## Materials and methods

### Tagging kites and demographic data

**Fig 1:**
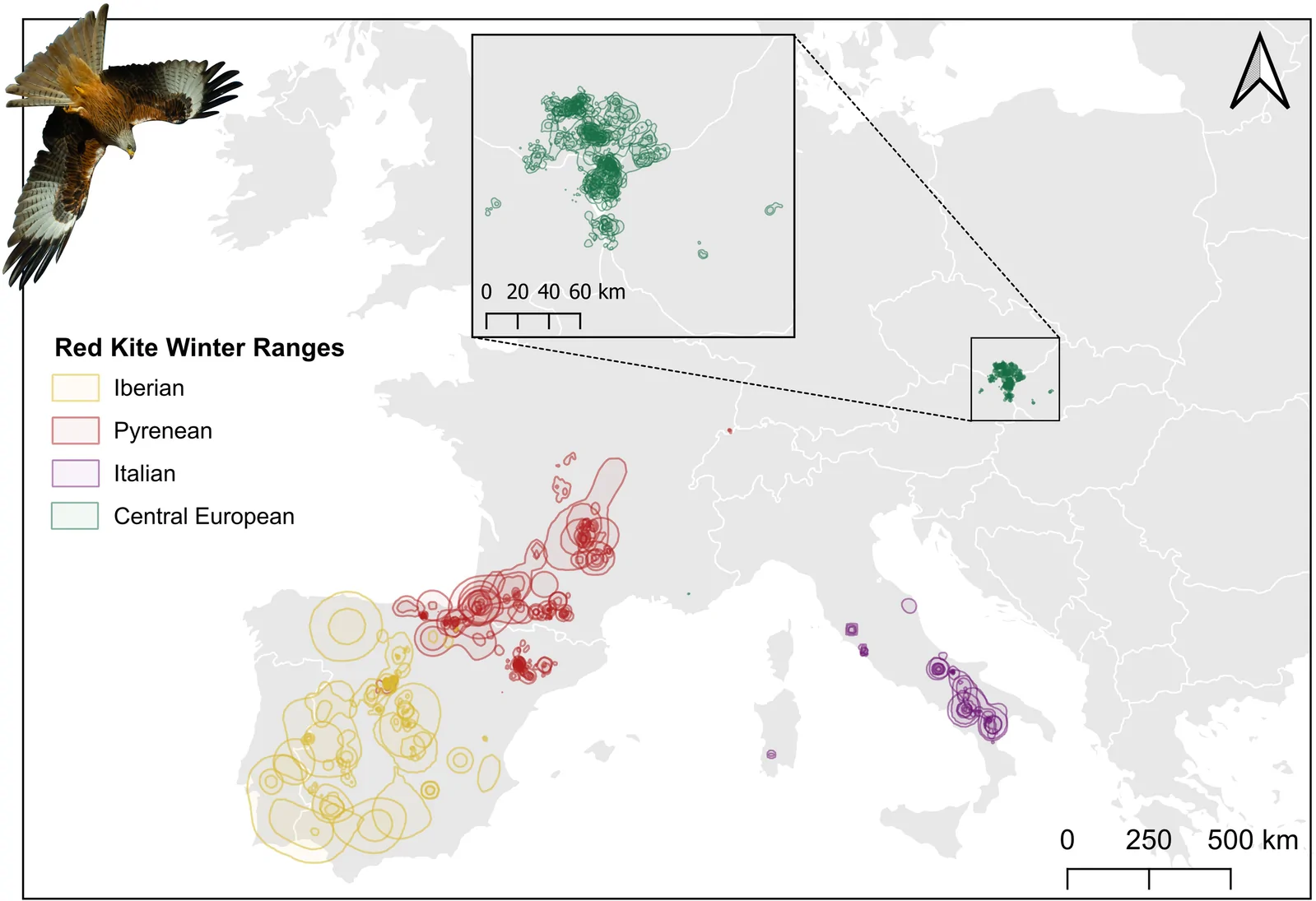
Spatial distribution of winter core areas (50% Kernel Density Estimators [KDE] utilisation distribution [UDs]) and home ranges (95% KDE UDs) for 103 GPS-GSM tracked Red Kites (*Milvus milvus*), over 180 winter periods, between 2013–2022. Inset map shows Central European kite winter ranges. Red Kite image republished under a CC BY license, with permission from Vicente Urios, original copyright 2026. Administrative boundary data derived from the Natural Earth Admin 0 Countries dataset (public domain).

This study was conducted in four European regions including Central Europe, Iberia, Italy, and the Pyrenees, which represent the main areas where kites tagged with GPS transmitters overwinter [56]. We classified individuals into four regional groups based on their wintering locations: Iberian (Spain and Portugal), Pyrenean (France and northern Spain), Italian, and Central European (Fig 1). These regional classifications are based solely on the discrete clusters of wintering locations of tagged kites and do not correspond to geopolitical boundaries or formal biogeographic regions. In total, 103 individual kites were included in the analyses presented in this study.

Between 2013–2022, wintering individuals were tagged in Spain (N = 42) and in Central Europe (N = 34). Although individuals were tagged only in Spain and Central Europe, tracked birds wintered across all four study regions (*i.e.*, Central Europe, Iberia, Italy, and the Pyrenees), and all individuals with sufficient winter tracking data were included irrespective of tagging location. Spanish birds were captured with clap nets shot from a distance by expert trappers at species-specific feeders in the provinces of Álava, Huesca and Segovia (69%, N = 29). The remaining birds (31%, N = 13) were captured with carrion set in their usual feeding areas in the provinces of Álava, Toledo and Segovia using the same capture system (Table 1) [30]. In Central Europe all birds were tagged as nestlings. Nestlings were temporarily removed from the nest and tagged at the end of their development at ages of approximately 35–40 days in Austria (N = 10), and the Czech Republic (N = 24). Handling and tagging of all birds took approximately 15 minutes per individual, with wintering birds being released at the same place of capture and the nestlings were returned to their nests.

**Table 1:** An overview of the tagging process, by country, for 103 wintering Red Kites (*Milvus milvus*) included in this study. Tagging activities were conducted in Austria, Czech Republic and Spain. In Austria and the Czech Republic, kites were tagged in the nest during the breeding season. In Spain, migrant kites were tagged during winter (see Methods for full details).

Tagging country	Estimated number of wintering individuals (winter periods surveyed)^§^	Age class when tagged				
		Nestling/pulli (%/total tagged)	First winter (%/total tagged)	Second winter (%/ total tagged)	Third winter or older (%/total tagged)	Total (%/total tagged)
Austria	203 (2019-2020)	10 (9.7)	0 (0)	0 (0)	0 (0)	10 (9.7)
Czech Republic	185 (2018-2019)	51 (49.5)	0 (0)	0 (0)	0 (0)	51 (49.5)
Spain	50,297 (2013-2014)	0 (0)	24 (23.3)	9 (8.7)	9 (8.7)	42 (40.8)
Total		61 (59.2)	24 (23.3)	9 (8.7)	9 (8.7)	103 (100)

§ Estimates based on winter roost count and road transect data published in Matsson *et al*. [56]. Original sources per country: Austria and the Czech Republic [57]; Spain [58].

Kites marked in Spain were tagged with solar-powered satellite, Solar Argos GPS or GPS-GSM devices: 8 gram – Solar PTT (N = 2, North Star, United States); OrniTrack-E25 (25 g, N = 22; Ornitela, Lithuania); 22 gram Solar Argos/GPS PTT (N = 2, North Star, United States); 21–22 gram Saker 4L, L and MH (N = 8; Ecotone, Gdynia, Poland). Kites marked in Central Europe were tagged with Saker H loggers (N = 53, 20 g, GPS/GSM; Ecotone, Poland) and OT-E25B-3G(C) loggers (N = 8, 25 g, GPS/GSM; Ornitela, Lithuania). In some cases, multiple devices were deployed across the study period (*e.g.*, due to replacement or re-tagging), and therefore the number of devices exceeds the number of individuals tracked.

On average, the loggers weighed approximately 2% of the kites’ body mass. We fitted loggers onto the back of the birds and secured them with Teflon ribbon (8.4–11.2 mm) using a thoracic harness encircling the body in two loops around the base of the wings and joining in front of the breastbone. In the case of Spain, all birds were marked with a harness that was released safely on an indicative basis at the end of the battery life of the transmitters [59]. Sex was determined by genetic analyses following the methodology proposed by Suh *et al*. [60]. We categorised each kite into distinct age classes: ‘first’ referring to all birds within their first winter (from hatching to the following spring), ‘immature’ included birds in their second winter (from the middle of the year following hatching until one year later), and ‘adult’, which included individuals within their third winter and older. We selected only year-round residents from Central Europe (N = 33) representing those that did not undergo short-to-medium distance migration. Those in all other regional categories (Pyrenees, Iberia and Italy) underwent migration; as birds tagged in Iberia and the Pyrenees were those that were tagged in winter and also included individuals wintering from other countries further north. Kites wintering in Italy were migrant individuals from Central Europe, where they were tagged as fledglings, but wintered in Italy. Therefore, adult breeding birds and local fledglings tagged in the Pyrenees, Iberia and Italy in spring are not considered in this study. We created a categorical variable term “migratory_status” and reclassified those wintering in Central Europe as “residents” (N = 37) and all other birds as “migratory” (N = 66) to explore the effects on PA overlap.

All tagging was performed under restrictions imposed by the co-author’s institutions. Permissions to deploy loggers onto kites was approved by Diputación Foral de Araba, Gobierno de Aragón, Inaga, Junta de Castilla y León, and Junta de Castilla-La Mancha.

### Winter kite core areas and home ranges

Winter core areas and home ranges were obtained from previously published GPS telemetry studies of kites wintering in Europe [26,40,48]. In these studies, arrival to and departure from the wintering grounds were determined based on marked shifts in daily travel distances and interruptions in latitudinal migration, with arrival defined as the point at which individuals ceased directional latitudinal movement and exhibited reduced daily travel distances. For each individual and winter, point kernel density estimates (KDEs) of space use were then generated from the GPS telemetry data [61] to estimate winter space-use metrics. We used these published winter KDEs to quantify overlap between kite winter ranges and protected areas. Point KDEs were selected in the original studies because they are widely used, intuitive, and facilitate comparisons with other research. However, KDE-based approaches have recognised limitations, including sensitivity to spatial autocorrelation in high-frequency tracking data and the potential to overestimate utilised areas [62]. Despite these limitations, KDEs remain widely used in movement ecology and were selected here to ensure comparability with previous studies of kite winter spatial ecology. Point KDEs produce utilisation distributions (UDs) that are probability density distributions in two-dimensional space, using relocations for each individual [63]. We used the standard metrics of 50% and 95% UDs of kite relocations interpreting these as “core areas” and “home ranges”, respectively. The relative performance of various smoothing parameters (bandwidth) estimators was explored, including the least squares cross-validation (LSCV) method [64]. The reference bandwidth was the most resilient to either over- or under-smoothing, and was therefore selected for all analyses. For each winter period, we obtained a core area polygon and a home range polygon based on the 50% and 95% UDs, respectively.

### Protected area and land cover data

To assess kite usage of European PA networks during the winter months, we downloaded the “Natura 2000” spatial data set (N = *c.* 27,000 individual sites; version 2021) from the European Environment Agency Datahub (https://www.eea.europa.eu/en/datahub) on 15 December 2023 in vector format. We also requested and downloaded all European “Important Bird and Biodiversity Area (IBA) digital boundaries” (N = 3,619 individual sites) March 2022 version from BirdLife International’s Datazone (https://datazone.birdlife.org) [65]. Using the “sf” package [66] in R version 4.3.2 [67], we projected all kite core area and home range polygons (in km^2^) into the WGS 1984 coordinate reference system. Then, we combined the Natura 2000 and IBA site shapefiles into a “PA” vector layer. We transformed both kite and PA polygons into the equal area European Grid coordinate reference system and performed an intersection analysis to calculate the proportional overlap (%) between the core areas and home range polygons, and the PA polygons.

We downloaded raster formatted land cover data on 1 January 2024 from the COPERNICUS Land Monitoring Service’s CORINE Land Cover 2018 database at 100-metre resolution [68]. Similarly, we downloaded the COPERNICUS Digital Elevation Model for Europe (EU-DEM) at 30-metre resolution derived from the COPERNICUS Global 30 Metres data set [69]. Using the “Reclassify by Table” function in QGIS version 3.14.16 [70], we reclassified the CORINE Land Cover data to create a new binary raster layer for agricultural land types only, i.e., all those listed under section “2. Agricultural areas”, which were assigned a value of 1. All other land cover types were assigned a value of 0. This classification reflects the importance of agricultural landscapes for winter foraging in kites and enables a focus on this key habitat type. For the purposes of this study, we defined “lowland” as topographical elevations ≤ 500 m ASL [26]. We also used the “Reclassify by Table” function to reclassify the raster EU-DEM layer to create a new binary layer for lowland raster cells (1) and non-lowland cells (0). To quantify associations between environmental variables and kite winter space use, we performed an intersection analysis in which we calculated the number of agricultural land cover and lowland cells within kite core area (50% UDs) and home range (95% UDs) polygons. To calculate proportional overlap, we divided the number of agricultural and lowland cells by the total number of cells within a polygon and multiplied by 100 resulting in the new variables “% agriculture” and “% lowland” which were used in subsequent statistical analyses.

### Statistical analyses

All statistical analyses were conducted in R version 4.3.2 [67]. We scaled and centred the following variables prior to inclusion in statistical models: core area size (km^2^), home range size (km^2^), % agriculture, and % lowland. In addition, we visually explored the goodness-of-fit distributions of the proportional “PA overlap (%)” variables, which revealed approximately Gaussian distributions. Therefore, to explore the effects of intrinsic and extrinsic variables on wintering kite use of European PAs, we ran a series of Linear Mixed Models (LMMs) using the “lme4” package [71] (see Table S1 in S1 File for a full description of variables used in this analysis). We ran full, global LMMs for kite core areas (50% UDs) and home ranges (95% UDs). For the full core area (50% UDs) model, proportional “PA overlap (%)” was fitted as the response variable with “sex” (factor: male or female), “age” (factor: first, immature or adult), “region” (factor: Central Europe, Iberia, Italy, or the Pyrenees), “migratory_status” (factor: migratory or resident), “core area size (km^2^)”, “% agriculture”, and “% lowland” fitted as explanatory variables. Model residuals were assessed for normality and heteroscedasticity. Previous research has shown that kites display high variability in post-reproductive movements between individuals [31], therefore, we accounted for this between-individual heterogeneity in space-use by fitting “bird_id” as a random effect. Initially, we also fitted “winter year” as a random effect but it did not explain any variation within our models and was therefore dropped from the final models. For the full home range (95% UDs) model, again proportional “PA overlap” (%) was fitted as the response variable, with “sex”, “age”, “region”, “migratory_status”, “home range size (km^2^)”, “% agriculture”, and “% lowland” fitted as explanatory variables. Similarly, “bird_id” was fitted as a random effect. We tested for multicollinearity between explanatory variables by examining the Variance Inflation Factors using the vif() function also in the “car” package [72], with values lower than 2.5 indicating no collinearity between fixed effects (Table S2 in S1 File) [73]. There was a significant correlation between “migratory_status” and “region”, therefore “migratory_status” was removed from the models and we retained “region”.

We applied an information-theoretic approach to rank and select the most appropriate core area (50% UDs) and home range (95%) model combinations. Automated model selection was performed using the dredge() function from the “MuMIn” package [74]. Model combinations were ranked according to their Akaike’s Information Criterion adjusted for small sample sizes (ΔAIC_c_) and associated model weights (Table 2). According to Burnham & Anderson [75], models were assumed “plausible” if the difference from the top model’s ΔAIC_c_ was < 4 and “substantial” if ΔAIC_c_ < 2 (Table 2). If more than one model provided substantial evidence, *i.e.*, ΔAIC_c_ < 2, models were averaged using the model.avg() function in the “MuMIn” package. Two core area models and four home range models provided substantial evidence and were therefore averaged (Table 2). As models were averaged using an AICc framework, inference is based on model-averaged coefficients and 95% confidence intervals; p-values are not reported.

**Table 2:** Model selection process explaining protected area overlap (measured in proportional overlap) by 103 GPS-GSM tracked wintering Red Kites (*Milvus milvus*) throughout their European range, between 2013 and 2022. Models presented for kite core areas (50% Kernel Density Estimator [KDE] utilisation distributions [UD]) and home ranges (95% KDE). Only models with an Akaike’s Information Criterion for small sample sizes (ΔAIC_c_) < 2 are presented. Those with a ΔAIC_c_ value < 2 provide substantial evidence (Burnham & Anderson 2002). Due to multiple models demonstrating substantial evidence for both model types (core areas and home ranges), we averaged the models presented and used them in the subsequent analyses. df = degrees of freedom.

Model	df	logLik	AIC_c_	ΔAIC_c_	Weight
*Core area (50% UD) models*					
age + % agriculture + core area size (km^2^) + % lowland + region + (1|bird_id)	11	−805.0	1633.6	0.00	0.49
age + % agriculture + % lowland + region + (1|bird_id)	10	−806.6	1634.4	0.84	0.32
*Home range (95% UD) models*					
age + % agriculture + home range size (km^2^) + % lowland + region + (1|bird_id)	11	−750.8	1525.1	0.00	0.24
age + % agriculture + % lowland + region + (1|bird_id)	10	−752.0	1525.2	0.16	0.22
age + % agriculture + home range size (km^2^) + region + (1|bird_id)	10	−752.4	1526.1	0.99	0.15
age + % agriculture + region + (1|bird_id)	9	−753.6	1526.3	1.17	0.13

## Results

### Overview of PA use by wintering kites

We compiled published winter core area and home range data for 103 kites, spanning 180 individual winter periods between 2013 and 2022 (Fig 1a–c). Each winter period corresponds to a single winter range estimate for an individual bird. Of the 103 individuals included in the analyses, 72 (69.9%) were sexed, with 36 (50%) female and 36 (50%) male, while 31 (30.1%) remained unsexed. Because many individuals contributed winter range estimates from multiple years, we calculated range estimates for 73 (40.6%) female winter ranges and 65 (36.1%) male winter ranges, with 42 (23.3%) winter ranges unable to be assigned to either sex. Of these, 76 (42.4%) represented adult ranges, 39 (21.7%) were from immature birds, and 65 (36.1%) were from first-year (juvenile) birds. Geographically, most winter ranges were in the Pyrenees (N = 78; 43.3%), followed by Central Europe (N = 44; 24.4%), Iberia (N = 37; 20.6%), and Italy (N = 21; 11.7%) (Fig 1a–c).

**Fig 2:**
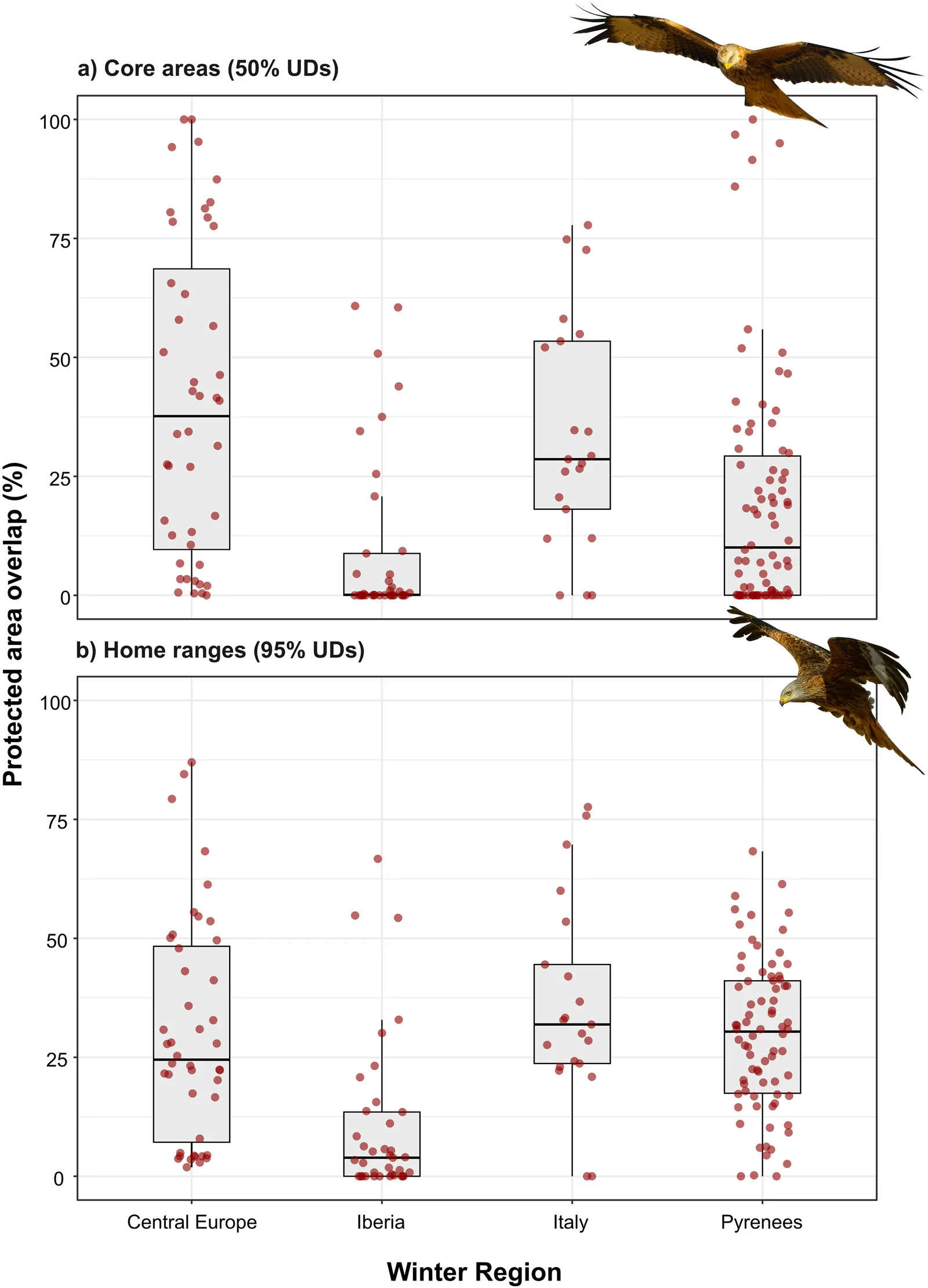
Protected area (PA) overlap (%) by 103 GPS-GSM tracked Red Kites (*Milvus milvus*) across their wintering regions, between 2013–2022. a) PA overlap (%) for kite core areas calculated using 50% Kernel Density Estimator utilisation distributions (KDE UDs) and b) PA overlap (%) for kite home ranges calculated using 95% KDE UDs. Red Kite images republished under a CC BY license, with permission from Vicente Urios, original copyright 2026.

On average, kite winter core areas overlapped European PAs by 24.5 ± 28.1% (standard deviation [SD]; range: 0–100%) and 26.6 ± 20.5% (0–87%) for kite winter home ranges. Along with this highly variable degree of overlap between individuals, there was large variation in winter kite range sizes across the data set, with mean sizes of 703 ± 2,581.9 km^2^ (0.1–29,549 km^2^) and 3,765.5 ± 12,390.1 km^2^ (1.7–127,513.5 km^2^) for kite core areas and home ranges, respectively (Fig 2a-c). Overlap with agricultural land cover averaged 66.3 ± 21% (4.7–100%) for kite core areas and 63.8 ± 19.6% (12.2–96.5%) for home ranges. Overlap with lowland averaged 67.5 ± 41.2% (0–100%) for kite core areas and 66.9 ± 38.2% (0–100%) for home ranges.

### Predictors of PA overlap within wintering kite core areas

**Fig 3:**
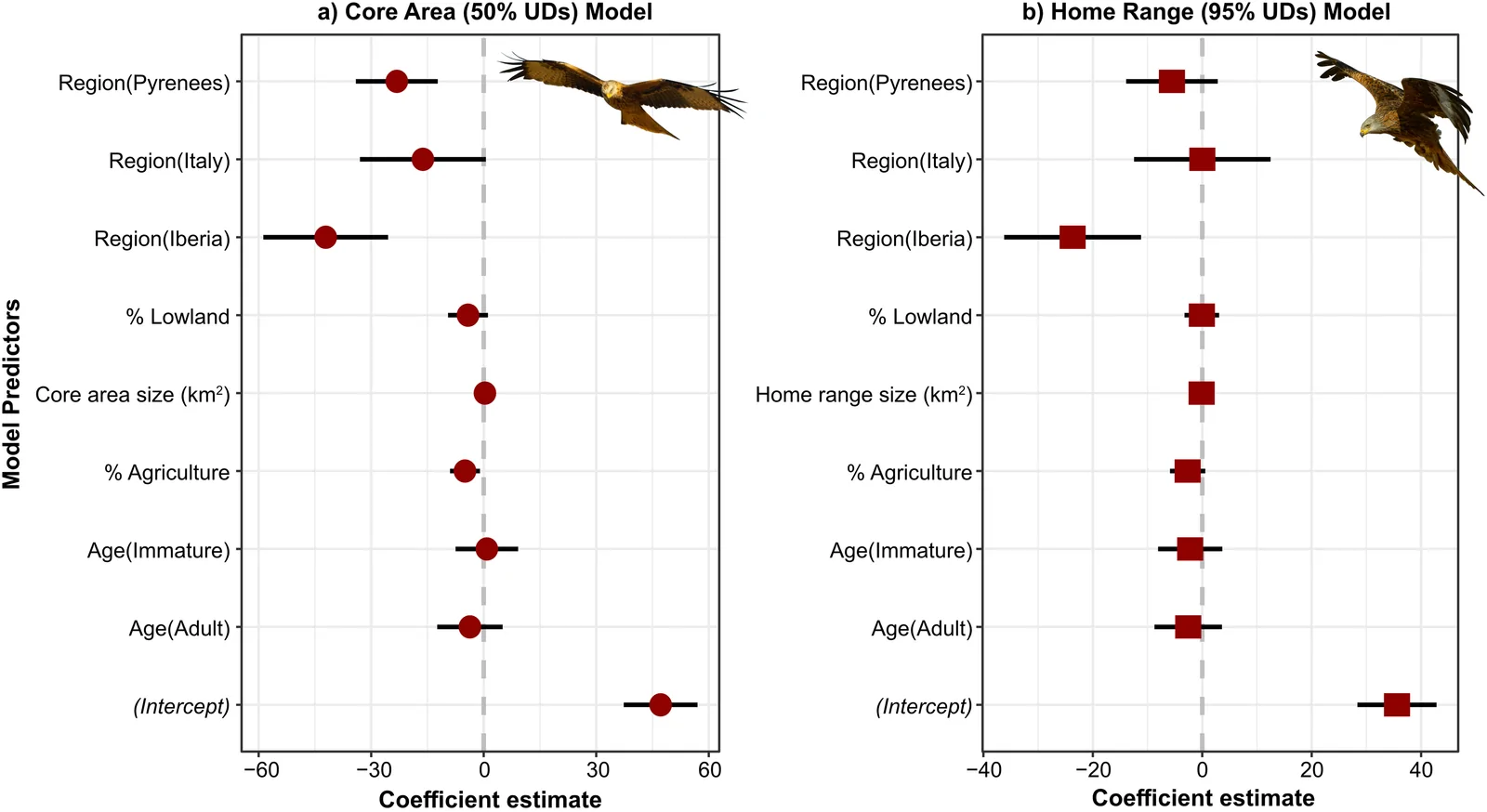
Modelled coefficient estimates, with 95% confidence intervals, explaining protected area (PA) overlap (measured as proportional overlap) by 103 GPS-GSM tracked wintering Red Kites (*Milvus milvus*) throughout their European range, between 2013–2022. a) estimates from the averaged core area model calculated using 50% Kernel Density Estimator utilisation distributions (KDE UDs) and b) the averaged home range model calculated using 95% KDE UDs. Reference groups: region = “Central Europe” and age = “first calendar year”. Note difference in x-axis scale. Red Kite images republished under a CC BY license, with permission from Vicente Urios, original copyright 2026.

From the averaged models (Table 2), kites that wintered in areas with greater proportional agricultural land cover showed a tendency to overlap PAs to a lesser extent (95% confidence interval [CI]: −8.98 to −0.99; Table S3 in S1 File; Fig 3a,b). Mean proportional PA overlap with kite core areas varied among regions (Fig 2a,b), with the highest overlap in Central Europe, followed by Italy, the Pyrenees, and Iberia. Region was the only strongly influential predictor of PA overlap, with kites wintering in the Pyrenees (95% CI: −34.08 to −12.25) and Iberia (−58.75 to −25.48) showing lower overlap compared to Central Europe (Table S3 in S1 File; Fig 3a,b). No differences were detected for Italy. Age, core area size (km^2^), and proportional lowland did not strongly influence PA overlap within kite winter core areas (Table S3 in S1 File; Fig 3a,b).

### Predictors of PA overlap within wintering kite home ranges

Mean proportional PA overlap across kite home ranges also varied among regions (Fig 2a,b), with the highest overlap in Central Europe and the lowest in Iberia. Region was the only influencing predictor of PA overlap, with kites wintering in Iberia showing lower overlap compared to Central Europe (95% CI: −36.21 to −11.19; Table S3 in S1 File; Fig 3a,b). There was no effect of proportional agriculture, age, home range size (km^2^), or proportional lowland on PA overlap (Table S3 in S1 File; Fig 3a,b). Sex was not an influential predictor in any model.

## Discussion

This is the first study to investigate the use of PAs by wintering kites in Europe. Kite winter ranges overlapped with PAs by approximately 25%, which is low given the extensive network of Natura 2000 and IBA sites across the European wintering range of the species [76]. Considering that PA coverage in Europe is approximately 18.6% [13], our findings suggest that overlap between kite winter ranges and PAs is modest relative to their broad-scale availability. However, we note that this interpretation is based on comparisons of proportional availability, and that formal resource selection approaches would be required to robustly assess preference or avoidance. Most notably, our data suggest that kites tend to winter in unprotected areas in Iberia, with mean proportional overlap between PAs and kite core areas and home ranges being 9.9% and 10.6%, respectively.

### Overlap with European PAs varies regionally and is affected by agricultural land

The most influential variables predicting overlap between kite wintering ranges and PAs were proportional agriculture and region. As expected, kites that wintered in areas with greater proportional agriculture had lower overlap with PAs than those that wintered in non-agricultural areas. Despite the Natura 2000 network covering all biogeographic regions in Europe [9], traditional agricultural landscapes often remain unprotected and tend not to be considered during designations of new PAs [53]. This likely reflects the historical prioritisation of semi-natural habitats and sites of high biodiversity value in PA designation, rather than human-modified landscapes. Our findings therefore highlight a potential mismatch between the spatial distribution of PAs and the habitats most frequently used by wintering kites. It is important to note that our analysis focuses on Natura 2000 and IBA networks, and does not include all nationally designated or IUCN-classified PAs. While this may result in some underestimation of total PA coverage, these networks represent the most spatially extensive and standardised conservation frameworks available for pan-European analyses of bird distributions.

Kites that spent the winter in Iberia and the Pyrenees used PAs to a lesser extent than conspecifics wintering in Central Europe. The relatively clustered distribution of Central European winter ranges likely reflects the spatial origin of the tracked birds, which originated from a single breeding population in the tri-border region of Austria, Slovakia and the Czech Republic. This population is known to winter predominantly within the Záhorská nížina Lowland and surrounding transboundary landscape, where communal roosting behaviour and regionally concentrated wintering habitat may contribute to the observed clustering of winter ranges [26]. These patterns could reflect greater use of protected areas by resident Iberian and Pyrenean kite populations during winter, or alternatively be driven by other extrinsic factors. For example, kites preferentially use agricultural landscapes, where prey such as voles (*Microtus* spp.) [77], and other anthropogenic food resources are often abundant, and these habitats are generally located outside protected areas. Use of anthropogenic waste sites, such as landfills and refuse dumps, by kites during winter has been reported within the literature [28,78,79], including in northern Iberia and the Pyrenees [80]. In southern Iberia, it has been reported that wintering kites have larger roosting areas, move further away from their roosting sites to feed and change feeding areas more frequently than resident kites [81]. Therefore, the fact that we only compared PA overlap between migrant kites from northern countries and thus non-residents may influence our results. Future studies should explore differences in the most important wintering areas, such as Iberia, in order to improve understanding of differences in winter space use between migrant and resident birds.

When natural food availability is limited, kites may also rely on anthropogenic food resources outside protected areas, including feeding stations [82]. More research is needed on how these food supplies and scavenging affect kite movements [83], especially with recent data on spatio-temporal variations in food availability [84]. Regional differences in PA overlap may also be influenced by factors such as social learning [85,86] and genetic variation among populations [87].

### Individual behavioural plasticity may explain non-influential age or sex effects

Despite age differences in winter range size being reported by previous research and a general preference towards lowland landscapes in winter [26,38], we found no relationships between kite age or proportional lowland on PA overlap. Kites exhibit substantial individual plasticity in their ranging behaviours, with multiple studies reporting notable between-individual differences in kite ranging behaviours during winter and also at other times of the year [31,38,48,88]. Such individual differences, driven by both extrinsic and intrinsic factors, may occur independently of age. Unlike other raptor groups such as bird-eating *Accipiter* hawks [89], kites show very little sexual dimorphism in size or plumage [90], which may explain why we found no influence of sex on PA overlap. Behavioural differences between the sexes are more pronounced during the breeding season when rearing young [38,43,91]. Consequently, pronounced sex differences driven by changes in kite energetic demands are evident during the breeding season, with male ranges being larger than female ranges [78,92], but less pronounced in winter with correspondingly reduced range size variation [26].

### A review of PA coverage that accounts for anthropogenic threats to kites is required

Our data suggest that existing PA networks provided limited coverage for kites during winter, particularly in Iberia and the Pyrenees. Importantly, although the species is listed under Annex I of the EU Birds Directive (Directive 2009/147/EC) and is therefore legally protected across its range, this legal protection does not necessarily translate into effective spatial coverage of key habitats used during winter. Furthermore, the effectiveness of PAs may depend on whether kites are explicitly recognised as a target species within site-specific management objectives. While Natura 2000 sites are designated to support species listed under the Birds Directive, management priorities and implementation can vary considerably among sites and regions. As such, areas used by wintering kites may not always be managed with this species as a focal conservation priority, which could limit the effectiveness of conservation actions even where spatial overlap occurs. Future work linking telemetry data with site-level management objectives would help to better understand how species-specific targeting influences conservation outcomes.

Working together with farmers, improved protection of these populations and important habitats for farmland biodiversity is urgently needed. Poisoning is a major threat to kite populations [32,33,35], leading to population declines [34] and limiting population recovery rates [93]. Many poisoning events are linked to pesticides and rodenticides, for example second generation anticoagulant rodenticides (AR) such as bromadiolone [94], used throughout intensively managed agricultural land [95,96]. High mortality rates due to secondary ARs within intensive arable agriculture has been reported in kite populations, with 70% of tested birds containing AR residues and 10% confirmed dead [97]. Use of ARs is widespread within commercial and intensive agricultural land due to the economic costs of rodent pests on crop yields [98]. Crucially, designation as a Natura 2000 site does not inherently prohibit agricultural activities, provided these do not adversely affect the conservation objectives for which the site was designated [99]. Similarly, IBAs do not themselves confer statutory legal protection, although they are widely used to identify sites of high conservation importance and may subsequently be designated under national or international conservation frameworks [15]. This highlights the need for targeted management actions, including measures to reduce exposure to rodenticides, to be explicitly integrated into PA management plans, particularly within key wintering regions. Preference for human-modified habitats outside of PAs may therefore increase exposure to anthropogenic threats and elevate the risk of human–wildlife conflict, especially in agricultural and other production landscapes [100].

### Study limitations may inhibit inference

Our use of KDEs to determine kite winter core areas and home ranges introduces known limitations, including sensitivity to spatial autocorrelation and the potential to overestimate utilised areas, particularly when using high-frequency GPS data [101,102]. Newer approaches such as adaptive local convex hulls are considered more biologically meaningful than KDEs [101–103]. However, we used KDEs to maximise the comparability of our findings with other studies of kite winter spatial ecology. Alternative approaches, such as autocorrelated KDEs or Brownian bridge movement models (BBMMs), may provide more refined estimates of space use and represent important avenues for future research [57,58,104]. Calculation of animal ranges using GPS relocations may also overestimate areas used by the individual [105]. Despite this, our use of solar-powered GPS-GSM satellite telemetry outperforms other methods to analyse kite space use, such as high frequency radio-telemetry, due to increased precision and accuracy while reducing sampling bias [106]. Kite overlap with PAs varied substantially within our data set, ranging from 0–100%, indicating substantial heterogeneity among individuals, which poses a challenge to conservation practitioners. Despite leveraging data from 103 individuals, we were unable to account for the spatial distribution of carrion [107], feeding stations [108], and natural prey availability [105], which likely contributed to a significant proportion of unaccounted heterogeneity across our averaged models. Future studies should analyse larger data sets to encompass individual behavioural variation and include more potentially influential variables to account for the “noise” in our models. These confounding factors may include spatio-temporal distribution of prey/carrion and rodenticide use, and micro-habitat variables affecting resource distribution and dispersion. Expanding the sample to include resident kites from the Iberian and Italian populations and other areas with increasing numbers of resident birds such as southern Sweden or large areas of central Europe [25] would improve our understanding of PA use in wintering kites. Furthermore, the legal protection status of IBA sites can vary between countries, and their ability to protect important habitats for biodiversity in law may therefore be limited [15]. In addition, even within formally designated PAs (e.g., Natura 2000), levels of protection and management effectiveness can vary considerably, with differences in permitted land use, enforcement, and conservation objectives potentially influencing their effectiveness.

### Conclusions and future research

Kite winter ranges overlapped with European Natura 2000 and IBA sites by approximately 25%, though this varied greatly. Such a relatively modest overlap, particularly in agricultural landscapes, suggests that current PA networks may not fully capture the spatial distribution of key wintering habitats for the species. Limited suitable winter habitat, such as traditional agricultural landscapes, might explain why kites use PAs less in Iberia and the Pyrenees. Kite populations in Spain are declining and poorly protected by existing areas. To address this, policymakers may consider broadening their focus to include biodiversity in human-modified environments. Collaboration with farmers and stakeholders is essential to improve protection for these populations and their habitats. Future research should examine: 1) the impact of food waste and carrion, 2) genetics and social learning, 3) habitat quality, 4) poisoning within protected areas, and 5) habitat or resource selection based on availability inside and outside of PA networks. Addressing threats to kite populations, especially in Iberia, is crucial, as understanding their space use and behavior is key to mitigating the effects of human activities on wildlife.

## Supporting information

S1 File Supporting tables for analyses of protected area usage by wintering Red Kites (*Milvus milvus*). Contains Table S1, summarising the variables included in the linear mixed-effects models; Table S2, reporting variance inflation factors (GVIFs) for fixed effects in candidate core area (50% KDE) and home range (95% KDE) models; and Table S3, reporting modelled 95% confidence intervals for fixed effects from the averaged linear mixed-effects models.
(DOCX)
