# Supplementary material for: Protected areas provide incomplete coverage for wintering Red Kites (Milvus milvus) in European agricultural landscapes
Source: PLoS One. 2026 Aug 26;21(8):e0355680. doi: 10.1371/journal.pone.0355680 (PMC13514066; doi:10.1371/journal.pone.0355680)
Supplement: Supplementary file 1 [file pone.0355680.s001.docx]

**SUPPLEMENTARY MATERIAL**

Protected areas provide incomplete coverage for wintering Red Kites (*Milvus milvus*) in European agricultural landscapes

**Table S1.** Summary of all variables included in the linear mixed-effects models used to explain protected area (PA) usage in 103 GPS-GSM tracked wintering Red Kites (*Milvus milvus*) throughout their European range between 2013 and 2022. For each variable, the table provides its role in the analyses, a brief description, units of measurement, and data provenance.

| Variable | Role in analysis | Model(s) | Type | Units / coding | Description | Provenance / source |
| --- | --- | --- | --- | --- | --- | --- |
| Protected area overlap | Response variable | Core area (50% UD); Home range (95% UD) | Continuous | % overlap (0–100) | Proportional overlap between each kite winter core area/home range polygon and the combined protected area layer. | Calculated in this study from published winter KDE polygons intersected with Natura 2000 and IBA spatial boundaries. |
| Sex | Fixed effect | Core area; Home range | Categorical | Male; female | Biological sex of each tracked kite. | Determined by genetic analyses following Suh *et al*. [58], as reported in the source telemetry studies. |
| Age class | Fixed effect | Core area; Home range | Categorical | First; immature; adult | Age class during winter. ‘First’ refers to the first winter after hatching, ‘immature’ to the second winter, and ‘adult’ to the third winter or older. | Derived from ringing/tagging information and categorised in the source telemetry studies. |
| Region | Fixed effect | Core area; Home range | Categorical | Central Europe; Iberia; Italy; Pyrenees | Regional group assigned according to wintering location. | Assigned from wintering locations of tracked birds in the compiled telemetry data. |
| Migratory status | Candidate fixed effect, removed before final models | Initially considered for Core area and Home range | Categorical | Resident; migratory | Classification of individuals as resident or migratory based on wintering region and movement behaviour. | Derived from tagging and wintering-location information in the compiled telemetry data. |
| Core area size | Fixed effect | Core area (50% UD) | Continuous | km²; scaled and centred for modelling | Size of each 50% utilisation distribution, interpreted as the winter core area. | Obtained from previously published GPS telemetry studies of wintering Red Kites [26,40,48]. |
| Home range size | Fixed effect | Home range (95% UD) | Continuous | km²; scaled and centred for modelling | Size of each 95% utilisation distribution, interpreted as the winter home range. | Obtained from previously published GPS telemetry studies of wintering Red Kites [26,40,48]. |
| Agricultural land cover within core area | Fixed effect | Core area (50% UD) | Continuous | % of 50% UD | Proportion of the winter core area classified as agricultural land. | Calculated in this study using CORINE Land Cover 2018 data at 100 m resolution. |
| Agricultural land cover within home range | Fixed effect | Home range (95% UD) | Continuous | % of 95% UD | Proportion of the winter home range classified as agricultural land. | Calculated in this study using CORINE Land Cover 2018 data at 100 m resolution. |
| Lowland within core area | Fixed effect | Core area (50% UD) | Continuous | % of 50% UD | Proportion of the winter core area occurring in lowland areas. | Calculated in this study using the Copernicus EU-DEM at 30 m resolution. |
| Lowland within home range | Fixed effect | Home range (95% UD) | Continuous | % of 95% UD | Proportion of the winter home range occurring in lowland areas. | Calculated in this study using the Copernicus EU-DEM at 30 m resolution. |
| Bird identity | Random effect | Core area; Home range | Categorical grouping variable | Individual identifier | Unique identifier for each tracked kite, used to account for repeated winter periods and between-individual heterogeneity. | Compiled from the source telemetry data. |
| Winter year | Candidate random effect, removed | Initially considered for Core area and Home range | Categorical grouping variable | Winter period / year | Winter period associated with each individual winter range. | Compiled from the source telemetry data. |

**Table S2.** Variance Inflation Factors (GVIF; tests of multicollinearity) between fixed effects of core area (50% Kernel Density Estimator utilisation distribution; KDE) and home range (95% KDE) model combinations, *i.e.,* those with Akaike’s Information Criterion adjusted for small sample sizes (ΔAIC_c_) < 2, prior to model averaging. Models explain protected area (PA) usage in 103 GPS-GSM tracked wintering Red Kites (*Milvus milvus*) throughout their European range, between 2013 and 2022 GVIF values close to 1 indicate no collinearity between fixed effects. df = degrees of freedom.

| Fixed effects | GVIF | df | GVIF(1/(2*df)) |
| --- | --- | --- | --- |
| *Core area model 1* | |  |  |
| age | 1.3 | 2 | 1.1 |
| region | 1.8 | 3 | 1.1 |
| core area size (km^2^) | 1.1 | 1 | 1.1 |
| % agriculture | 1.2 | 1 | 1.1 |
| % lowland | 1.7 | 1 | 1.3 |
| *Core area model 2* | |  |  |
| age | 1.2 | 2 | 1.0 |
| region | 1.7 | 3 | 1.1 |
| % agriculture | 1.2 | 1 | 1.1 |
| % lowland | 1.7 | 1 | 1.3 |
| *Home range model 1* | |  |  |
| age | 1.3 | 2 | 1.1 |
| region | 2.0 | 3 | 1.1 |
| home range size (km^2^) | 1.2 | 1 | 1.1 |
| % agriculture | 1.3 | 1 | 1.2 |
| % lowland | 1.8 | 1 | 1.3 |
| *Home range model 2* | |  |  |
| age | 1.2 | 2 | 1.0 |
| region | 1.9 | 3 | 1.1 |
| % agriculture | 1.3 | 1 | 1.1 |
| % lowland | 1.8 | 1 | 1.3 |
| *Home range model 3* | |  |  |
| age | 1.2 | 2 | 1.1 |
| region | 1.4 | 3 | 1.1 |
| % agriculture | 1.3 | 1 | 1.1 |
| home range size (km^2^) | 1.2 | 1 | 1.1 |
| *Home range model 4* | |  |  |
| age | 1.1 | 2 | 1.0 |
| % agriculture | 1.2 | 1 | 1.1 |
| region | 1.3 | 3 | 1.1 |

**Table S3**. Modelled 95% confidence intervals (CIs) for fixed effects from the averaged Linear Mixed models explaining protected area usage in 103 GPS-GSM tracked wintering Red Kites (*Milvus milvus*) throughout their European range, between 2013 and 2022. ‘50% Core area model’ refers to protected area (PA) use throughout kite core areas calculated using 50% Kernel Density Estimator (KDE) utilisation distributions and ‘95% UD model’ refers to PA use throughout kite home ranges based on 95% KDEs. Reference levels for categorical variables include: age = “first calendar year” and region = “Central Europe”. SE = standard error. Variables that were influential (those with CIs that did not overlap zero) are shown in **bold**.

| Fixed effect | 95% CI | |
| --- | --- | --- |
|  | 2.5% | 97.5% |
| *50% Core area model* |  |  |
| **(Intercept)** | **37.32** | **57.00** |
| age(immature) | -7.51 | 9.18 |
| age(adult) | -12.40 | 5.51 |
| **region(Iberia)** | **-58.75** | **-25.48** |
| region(Italy) | -33.00 | 0.59 |
| **region(Pyrenees)** | **-34.08** | **-12.25** |
| core area size (km^2^) | -3.03 | 4.11 |
| **% agriculture** | **-8.98** | **-0.99** |
| % lowland | -9.51 | 1.13 |
| *95% Home range model* |  |  |
| **(Intercept)** | **28.36** | **42.83** |
| age(immature) | -8.08 | 3.67 |
| age(adult) | -8.73 | 3.61 |
| **region(Iberia)** | **-36.21** | **-11.19** |
| region(Italy) | -12.47 | 12.49 |
| region(Pyrenees) | -13.92 | 2.84 |
| home range size (km^2^) | -2.84 | 2.40 |
| % agriculture | -5.90 | 0.56 |
| % lowland | -4.14 | 3.89 |
